# A case of Reversible Dementia associated with Sero‐negative Autoimmune Encephalitis‐ A case study

**DOI:** 10.1002/alz70857_102104

**Published:** 2025-12-25

**Authors:** Praveen Nandha Kumar Pitchan Velammal, Sathish Kumar Mani, Nirumal Khumar, Amara Karteek, Aishwarya Jaikrishnan

**Affiliations:** ^1^ Tirunelveli Medical College, Tirunelveli, Tirunelveli, TamilNadu, India; ^2^ Saveetha Medical College and Hospital, Chennai, Chennai, TamilNadu, India; ^3^ Saveetha Medical College and Hospital, Chennai, TamilNadu, India

## Abstract

**Background:**

Giant Cell Arteritis (GCA) typically presents with unilateral headaches, jaw claudication, and scalp tenderness, often treated with corticosteroids. However, long‐term steroid use can cause neuropsychiatric symptoms and infections. This case involves a 60‐year‐old diabetic female with GCA who initially improved with methylprednisolone but later developed encephalopathy and neuropsychiatric symptoms during steroid tapering. Despite a negative autoimmune encephalitis panel, her response to intravenous immunoglobulin (IVIG) suggests an autoimmune component.

**Method:**

Case study

**Results:**

A 60‐year‐old diabetic female presented with severe, unilateral right‐sided headaches, jaw claudication, scalp tenderness, and small joint pain for three months. Giant Cell Arteritis (GCA) was suspected and initially treated with methylprednisolone, leading to significant improvement. Three months later, she developed high‐grade fever, altered sensorium, irritability, and confusion. Investigations revealed an elevated CRP (314 mg/L) and ESR (114 mm/hr), while MRI showed gliosis in the right temporal lobe. CSF analysis was normal, and Klebsiella pneumoniae was isolated from urine. Encephalopathy secondary to UTI was suspected. Empiric treatment with antibiotics and antivirals improved her symptoms. After 3 months, she presented with paranoia, agitation, and tremulousness while tapering steroids. CT and vasculitis workups were negative. Given concerns over steroid‐induced myopathy and possible autoimmune encephalitis, she received intravenous immunoglobulin (IVIG) therapy, resulting in substantial clinical improvement. The patient was discharged on oral prednisone, azathioprine, statins, antiplatelets, and levetiracetam. Although the autoimmune encephalitis panel was negative, the patient's response to immunomodulatory therapy suggests an autoimmune component. This case highlights the diagnostic challenge in recurrent altered sensorium in GCA and the importance of considering autoimmune or steroid‐induced etiologies in such patients.

**Conclusion:**

This case highlights the diagnostic challenge in GCA patients with recurrent neuropsychiatric symptoms. It emphasizes the need to consider steroid‐induced effects and autoimmune etiologies, as the patient's response to IVIG points to a possible autoimmune component despite negative tests.

